# Kidney Biopsy in Cancer Patients With Acute Kidney Disease: Histological Findings and Kidney Outcomes

**DOI:** 10.7759/cureus.105813

**Published:** 2026-03-25

**Authors:** Christina Kaitantzoglou, Ioanna Revela, Ioannis Giatras, Niki Markou, Stavros Fokas, Charikleia Gakiopoulou, Alexandros Gerakis

**Affiliations:** 1 Nephrology Department, Hygeia Hospital, Athens, GRC; 2 Nephrology Department, Frontis Dialysis Center, Athens, GRC; 3 First Department of Pathology, School of Medicine, National and Kapodistrian University of Athens, Athens, GRC

**Keywords:** acute interstitial nephritis, acute kidney injury, cancer, immunotherapy, kidney biopsy, nephrotoxicity, onconephrology, systemic anticancer therapies, targeted therapy

## Abstract

Background and aims

The incidence and prevalence of acute kidney disease (AKD), including acute kidney injury (AKI) and proteinuria, are increased in cancer patients. This study aimed to investigate the variety of renal histopathological lesions in cancer patients and their associations with systemic anticancer therapies (SACT) before and after 2017, the year when new SACTs like immune checkpoint inhibitors and target therapies were generally introduced, as well as the role of kidney biopsy (KB) in guiding treatment decisions.

Material and methods

This retrospective cohort study included adult cancer patients who underwent kidney biopsy for AKD at a single center over a 14-year period. Clinical, laboratory, and renal histopathological data were collected, and therapeutic options were evaluated. The study cohort was stratified into three groups - (i) SACT-related AKD, (ii) cancer-related AKD, and (iii) non-SACT/non-cancer-related AKD - based on clinical judgment by the treating nephrologist, allowing detailed characterization of AKD patterns.

Results

Fifty-five patients (61.8% men; mean age 64.7±9.9 years) were included. At diagnosis, median serum creatinine was 2.2 mg/dl (interquartile range (IQR) 1.6-4) and proteinuria 1.3 g/24h (IQR 0.25-4). Most patients (63.6%) were receiving SACT. Glomerulopathies were the most frequent lesions (43.6%), with focal segmental glomerulosclerosis (FSGS) predominating (29%). Acute interstitial nephritis (AIN), acute tubular injury (ATI), and mixed AIN/ATI were identified in 21.8%, 12.7%, and 9.1% of cases, respectively, while renal tumor infiltration occurred in 7.3%. AKD was attributed to SACT in 41.8%, most commonly immunotherapy (52.2%), and AIN was the prevailing lesion (82.6%). A significant shift in histopathological patterns was observed after 2017 (p=0.006), with higher frequencies of glomerulonephritis (51.3% vs. 25%) and acute tubulointerstitial nephritis (25.6% vs. 12.5%). Among patients receiving targeted treatment based on KB findings, including corticosteroid therapy, all experienced favorable renal outcomes.

Conclusions

This study highlights the expanding spectrum of kidney injury in cancer patients, the influence of modern SACT on renal pathology, and the critical role of kidney biopsy in guiding individualized management strategies.

## Introduction

Cancer patients have an increased risk for acute kidney disease (AKD), reported to be approximately 17% within one year after diagnosis [1]. This may be related to the nephrotoxic potential of anticancer therapies and the presence of comorbidities. Additional contributing factors include dehydration, infections, exposure to iodinated contrast media, and other nephrotoxic agents. Moreover, kidney injury may result directly from the malignancy itself through mechanisms such as urinary tract obstruction, tumor lysis syndrome, infiltration of neoplastic cells, hypercalcemia, and paraneoplastic manifestations, including glomerulopathies [2].

Consequently, managing a cancer patient with AKD is often challenging and an onco-nephrologist should estimate the potential influence of systemic anticancer therapies (SACT) on AKD.

Until recent years, kidney biopsies were rarely performed on cancer patients experiencing AKD, leading to a scarcity of histological data in this population [3]. This was related to several factors. First, these patients had high morbidity and poor prognosis, with a two-fold elevated mortality compared to those without AKD, as shown by published data [3]. This may be due to the inability to administer the optimal dose of anticancer therapy, participate in therapeutic protocols, or undergo necessary imaging tests [4]. Secondly, the renal injury patterns of conventional SACT were relatively well characterized. Acute tubular necrosis, electrolyte disorders, membranous nephropathy and thrombotic microangiopathy-haemolytic uremic syndrome (TMA-HUS) were reported as the most common types of renal injury [5]. Kidney injury was typically evaluated based on clinical grounds, laboratory blood and urine tests.

However, the oncology landscape has changed, as less toxic and more promising therapies have emerged, including targeted therapy and immune checkpoint inhibitors (ICI). Patients’ survival has improved, with five-year survival rates for all cancer types increasing from 50% in the mid-1970s to 70% in 2021 [6]. Despite this fact, the incidence of nephrotoxicity has not been decreasing [7].

Targeted therapy is a novel cancer therapy based on interventions that target the tumor’s abnormal growth process [8]. These are small molecules or larger autoantibodies. The commonest pattern of kidney injury in tyrosine kinase inhibitor (TKI) toxicity is podocytopathy, but membranous nephropathy (MN) and tubulointerstitial nephritis have also been reported. The main renal side effects of antiangiogenic agents are microangiopathy and podocytopathy [9].

ICIs are monoclonal antibodies against two surface receptors: cytotoxic T lymphocyte-associated antigen 4 (CTLA-4) and programmed death-1 (PD-1) are located on T-cells and natural killer cells and the latter's ligand, programmed death ligand-1 (PDL-1) can be seen on tumor cells [10]. Treating cancer patients with these agents aims at triggering the immune system to attack cancer cells [11]. This may lead to autoimmunity and a broad spectrum of immune-related adverse events (irAEs) [10,11]. ICI administration may cause kidney injury (ICI-associated AKI (ICPI-AKI)), with an estimated incidence of 1%-2% [12]. The most common pattern of renal injury is acute interstitial nephritis (AIN) (93%) [11]. However, many other histological lesions can be observed.

Moreover, the clinicopathological expression of renal injury has changed; AIN appears to be more frequent in comparison to former years, while rare histological findings [13] are now reported as well. If we also take into account the increase in these patients’ survival, the indication for kidney biopsy is proposed to be similar to that of the general population [14]. This fact has led to an increase in kidney biopsies performed on cancer patients with AKD over the last five years [14-16].

In this retrospective study, we aim to assess the spectrum of renal histopathological findings in cancer patients and their association with the received SACT. We also assessed whether there was a shift in histological patterns after 2017 as a counter to the toxicity of ICIs and targeted therapy. In addition, we assess the utility of kidney biopsy in guiding decisions regarding continuation of anticancer therapy and selection of appropriate treatment.

## Materials and methods

Study design and methods

This single-center retrospective study included adult patients with solid or hematologic malignancies who underwent kidney biopsy for AKD between September 2010 and February 2024. The criteria for performing a kidney biopsy in these patients included unexplained acute kidney disease, manifested as acute kidney injury (defined as an increase in serum creatinine >1.5 times the baseline value or a reduction in urine output), and/or evidence of kidney damage, such as proteinuria and/or an active urinary sediment. All biopsies were performed after cancer diagnosis. Demographic, clinical, laboratory, and renal histopathological data were extracted from electronic health records. Kidney function was assessed using the Chronic Kidney Disease Epidemiology Collaboration equation (CKD-EPI) [17]. AKD was defined according to the Kidney Disease Improving Global Outcomes (KDIGO) criteria [18] and encompasses acute kidney injury (AKI), a decline in eGFR, and/or evidence of kidney damage, such as new or worsening proteinuria. Renal recovery was classified as complete (serum creatinine 1.5× baseline without need for hemodialysis), or no recovery (persistent requirement for renal replacement therapy). Concerning glomerulonephritis, recovery was evaluated on laboratory grounds, including resolution of active urine sediment and proteinuria, as well as improvement in renal function. Follow-up started at the time of AKD diagnosis and continued until the study was completed. A favorable renal outcome was defined as complete recovery, remission of glomerulonephritis, or partial recovery without dialysis for at least three months. The year 2017 was selected as the treatment-period cutoff, reflecting the introduction of new SACT, namely ICIs and targeted therapies, in our hospital’s daily practice. To examine the association between renal histopathological patterns and AKD-related SACT and to assess differences in SACT initiation before and after 2017, the cohort was stratified into three groups: SACT-related AKD, cancer-related AKD, and non-SACT/non-cancer-related AKD. The classification was based on the treating nephrologist's clinical judgment rather than formal statistical criteria, enabling detailed characterization of AKD patterns in the context of SACT exposure and underlying malignancy.

Statistical analysis

The results are expressed as mean±standard deviation (SD) for normally distributed continuous variables, as median with interquartile range (IQR) for skewed variables and as number (N) and percentage (%) for categorical variables. Continuous variables were tested for normal distribution using the Kolmogorov-Smirnov test. Comparisons between groups were studied with Student’s t test for normally distributed variables; otherwise Μann-Whitney U test was applied. Categorical data were compared by Chi-square test or Fisher’s exact test, where appropriate. A two-sided p-value <0.05 was considered statistically significant. All analyses were performed with the statistical software package STATA version 13.0 (Stata Corp., College Station, TX, USA).

## Results

Baseline patients’ characteristics

A total of 55 patients underwent kidney biopsy (KB), including 34 (61.8%) men with a mean age of 64.7±9.9 years. Table 1 shows clinical characteristics, malignancy diagnoses, and laboratory values at KB for all patients.

**Table 1:** Patients’ characteristics at kidney biopsy (N=55) Continuous variables are presented as mean±SD or median (IQR) depending on distribution, and categorical variables as N (%). CKD: Chronic kidney disease; SD: standard deviation; AL: amyloid light chain

Parameter	N=55
Age, years (SD)	64.7 (9.9)
Gender (male)	34 (61.8)
Hypertension	14 (25.4)
Diabetes mellitus	10 (18.1)
CKD	5 (9.1)
Creatitine, mg/dl^#^	2.2 (1.6-4)
eGFR (ml/min/1.73m^2^)^#^	27 (12-45)
Proteinuria, g/24h^#^	1.3 (0.25-4)
Follow-up, months^#^	12 (5-38)
Malignancies	
Solid tumors	39 (70,9)
Lung	13 (33.3)
Gastrointestinal	5 (12.8)
Breast	4 (10.2)
Sarcomas	4 (10.2)
Melanoma	3 (7.7)
Kidney	3 (7.7)
Urinary tract	2 (5.1)
Endometrial	2 (5.1)
Hepatic	1 (2.6)
Glioblastoma	1 (2.6)
Mesothelioma	1 (2.6)
Hematological	16 (29.1)
Multiple Myeloma	6 (37.5)
Lymphomas	5 (31.2)
Lymphoblastic leukemia acute/chronic	3 (18.8)
AL Amyloidosis	2 (12.5)

The median follow-up was 12 months (IQR, 5-38 months). At KB, the median serum creatinine was 2.2 mg/dL (IQR, 1.6-4.0), eGFR was 27 mL/min/1.73 m² (IQR, 12-45), and proteinuria was 1.3 g/24 h (IQR, 0.25-4.0). Among patients with solid tumors (70.9%), lung cancer was most common (33.3%), followed by gastrointestinal cancers (12.8%). In hematological malignancies, multiple myeloma (37.5%) and lymphomas (31.2%) were most frequent. The preexisting chronic kidney disease was present in 9.1% of patients, hypertension in 25.4%, and diabetes mellitus in 18.1%. The main reason for KB was acute kidney injury (AKI) (51%), followed by combined AKI and proteinuria (38%) and isolated proteinuria (11%). Half of the patients with proteinuria had nephrotic-range proteinuria.

When kidney injury occurred, the majority of patients (n=35, 63.6%) were receiving SACT: 28.6% (n=10) on chemotherapy, 25.7% (n=9) on ICI, 8.6% (n=3) on targeted therapy, 17.2% (n=6) on combined ICI and chemotherapy, 8.6% (n=3) on combined ICI and targeted therapy, 2.8% (n=1) on hormone therapy, 5.7% (n=2) on monoclonal antibodies, and 2.8% (n=1) on a proteasome inhibitor (Table 2).

**Table 2:** Characteristics of acute kidney injury (AKI) and proteinuria according to systemic anticancer therapy (SACT). Data are presented as number of patients (N) and percentage (%). AKI staging (1–3) and proteinuria grading (A1–A3) were defined according to KDIGO criteria [18, 19]. Abbreviations: AKI: acute kidney injury, SACT: systemic anticancer therapy, ICI: immune checkpoint inhibitor, TKI: tyrosine kinase inhibitor, anti-VEGF: anti vascular endothelial growth factor

SACT	N=35	AKI, N (%)	Stage of AKI* (per patient)				Proteinuria, N (%)	Proteinuria** (per patient)		AKI + Proteinuria, N (%)	Stage of AKI/grade of proteinuria (per patient)	
Immune checkpoint inhibitor (ICI)	9 (25.7)											
Pembrolizumab	4 (11.4)	1 (2.9)	2				2 (5.7)	A3	A3	1 (2.9)	1/A2	
Nivolumab	1 (2.9)	0 (0)	-				0 (0)	-		1 (2.9)	1/A2	
Ipilimumab**	1 (2.9)	1 (2.9)	2				0 (0)	-		0 (0)	-	
Dual (ipilimumab+nivolumab)	3 (8.6)	3 (8.6)	2	2		1	0 (0)	-		0 (0)	-	
ICI+ chemotherapy	6 (17.1)											
Pembrolizumab+pemetrexide	2 (5.7)	2 (5.7)	2	2			0 (0)	-		0 (0)	-	
Pembrolizumab+carboplatin	4 (11.4)	2 (5.7)	3	2			1 (2.9)	A2		1 (2.9)	2/A2	
ICI+ TKI	3 (8.6)											
Pembrolizumab+axitinib	3 (8.6)	2 (5.7)	1	1			0 (0)	-		1 (2.9)	1/A2	
Chemotherapy	10 (28.6)											
Methotrexate	1 (2.9)	1 (2.9)	2				0 (0)	-		0 (0)	-	
Doxorubicin	2 (5.7)	1 (2.9)	1				0 (0)	-		1 (2.9)	2/A3	
Folic acid-5FU-irinotecan	1 (2.9)	0 (0)	-				0 (0)	-		1 (2.9)	2/A2	
Pemetrexide+carboplatin	1 (2.9)	0 (0)	-				0 (0)	-		1 (2.9)	3/A3	
Ifosfamide	5 (14.3)	3 (8.6)	2	3	3		0 (0)	-		2 (5.7)	2/A3	2/A2
Targeted therapy	4 (11.4)											
TKIS (ibrutinib, pazopanib)	2 (5.7)	0 (0)	-				0 (0)	-		2 (5.7)	2/1	1/A2
Αnti-VEGF (bevasizumab)	1 (2.9)	0 (0)	-				1 (2.9)	A3		0 (0)	-	
Aromatase inhibitor (aromacin)	1 (2.9)	1 (2.9)	1				0 (0)	-		0 (0)	-	
Monoclonal antibodies	2 (5.7)											
Daratumumab	1 (2.9)	0 (0)	-				0 (0)	-		1 (2.9)	2/A2	
Rituximab	1 (2.9)	1 (2.9)	2				0 (0)	-		0 (0)	-	
Proteasome inhibitor	1 (2.9)											
Vortezomibe	1 (2.9)	0 (0)	-				0 (0)	-		1 (2.9)	2/A2	

The median time from SACT initiation to AKD is reported as nine months, with a range of 6-18 months. The remaining patients (n=20) were not receiving SACT at the time of AKD, or had discontinued SACT due to related adverse events.

Renal histopathological findings

**Figure 1:**
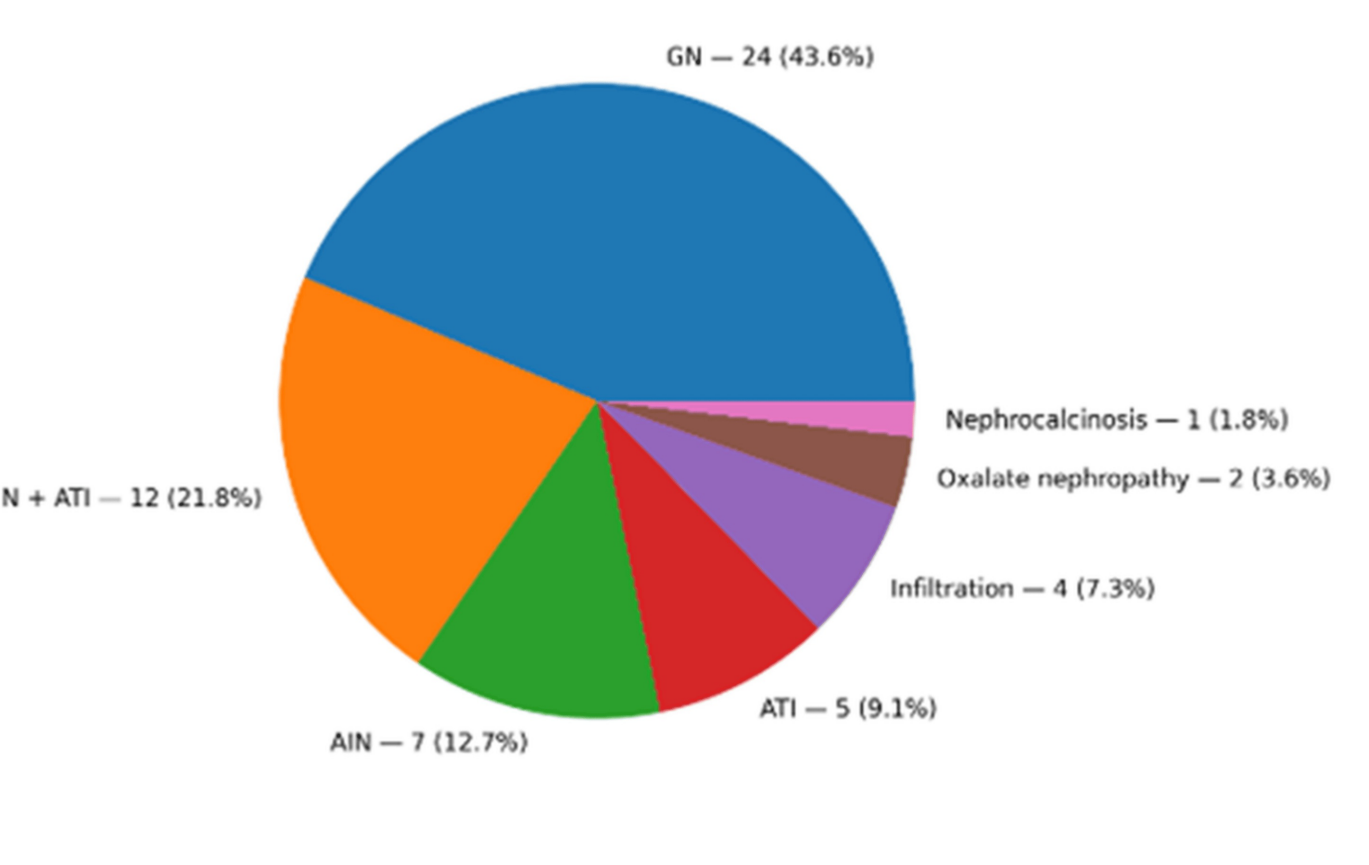
Distribution of kidney biopsy diagnoses among patients with cancer and acute kidney disease (N = 55) Data are presented as number of patients (N) and percentage (%). AIN: acute interstitial nephritis; ATI: acute tubular injury; GN: glomerulonephritis.

Glomerulopathy was the most common diagnosis (43.6%, n=24, Figure 1).

**Figure 2:**
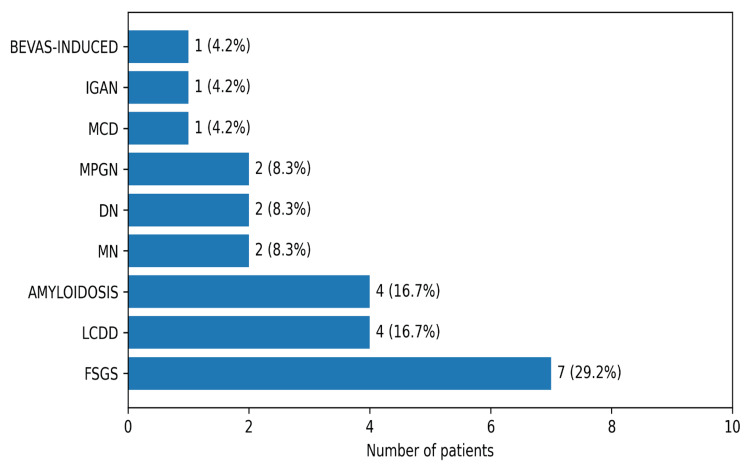
Spectrum of glomerular diseases identified on kidney biopsy (N=24) Data are presented as number of patients (N) and percentage (%). Abbreviations: FSGS: Focal segmental glomerulosclerosis, LCDD: Light chain deposit disease, MN: membranous nephropathy, DN: diabetic nephropathy, MPGN: membranoproliferative glomerulonephritis, MCD: minimal change disease, IGAN: IgA nephropathy, BEVAS-INDUCED: Bevasizumab-induced hyaline occlusive glomerular microangiopathy.

Focal segmental glomerulosclerosis (FSGS) was the most frequent lesion (29%), followed by light chain deposit disease (LCDD) (16.7%), amyloidosis (16.7%), and MN (8.3%) (Figure 2).

Amyloidosis was present in 16.6% of patients, including three cases of immunoglobulin light chain (AL) subtype and one of serum amyloid A (AA) subtype. Single cases included MN with full-house immunofluorescence and hyaline occlusive glomerular microangiopathy. Other lesions involved minimal change disease (MCD), IgA nephropathy, diabetic nephropathy, and membranoproliferative glomerulonephritis (MPGN) (Figure 2). AIN was identified in 21.8% (n=12), ATI in 12.7% (n=7), and a combination of both (AIN+ATI) in 9.1% (n=5) of patients (Figure 1). Notably, 75% of patients treated with ifosfamide for sarcoma developed karyomegalic tubulointerstitial nephritis.

Renal infiltration by tumor cells was observed in 7.3% (n=4) of the patients, including three cases of B-cell lymphoma and one case of urothelial carcinoma. Additionally, oxalate nephropathy was identified in 3.6% (n=2), and nephrocalcinosis in 1.9% (n=1) of patients (Figure 1).

Renal histopathological patterns in SACT-related AKD, cancer-related AKD, and non-SACT/non-cancer-related AKD

**Figure 3:**
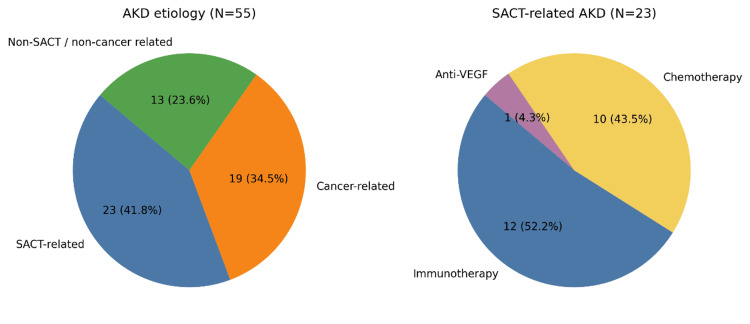
Classification of acute kidney disease (AKD) according to etiology (SACT-related, cancer-related, or unrelated) and cancer treatment types between 2010–2024. Data are presented as N (%).

To describe renal histological patterns in cancer patients with AKD, the study population was categorized into three groups: SACT-related AKD, cancer-related AKD, and non-SACT/non-cancer-related AKD (Figure 3).

Classification was determined by the treating nephrologist based on clinical and laboratory assessment, enabling detailed characterization of AKD patterns across SACT exposure and underlying malignancy.

SACT-related AKD was observed in 41.8% of patients (n=23). Most received ICIs (52.2%), followed by chemotherapy (43.5%) and bevacizumab (4.3%) (Figure 3). The main lesion was AIN (n=19, 82.6%), while 17.4% (n=4) had glomerular diseases, including MN with full-house immunofluorescence, AA amyloidosis, cellular variant FSGS, and hyaline occlusive glomerular microangiopathy. All patients discontinued SACT, with 34.8% (n=8) stopping temporarily and 65% (n=15) permanently.

Cancer-related AKD was seen in 34.6% of patients (n=19), with findings such as tumor cell infiltration (n=4), secondary glomerulopathies, and cancer-associated conditions like LCDD (n=4), FSGS (n=3), AL amyloidosis (n=2), membranous nephropathy (n=1), MPGN (n=1), MCD (n=1), ATI (n=2), and oxalate nephropathy (n=1). Patients in this group had more comorbidities, with hypertension and diabetes present in 42% compared to 26% in the SACT-related AKD group (Table 3). 

**Table 3:** Cancer-related acute kidney disease group: Histological lesions and comorbidities. Clinical and pathological characteristics are presented descriptively for each patient. Abbreviations: NHL: Non-Hodgkin’s lymphoma, MPGN: membranoproliferative glomerulopathy, ATI: acute tubular necrosis, MM: multiple myeloma, AIN: acute interstitial nephritis, FSGS: focal segmental glomerulosclerosis, DM: diabetes mellitus, MN: membranous nephropathy, MCD: minimal change disease, MGUS: monoclonal gammopathy of undetermined significance, LCDD: light chain deposition disease, CLL: chronic lymphocytic leukemia

Patient	Cancer	Histological lesion	Comorbidities
1	NHL	MPGN	Cryoglobulinemia
2	Pancreas	ATI	Diarrhea
3	MM	AIN	none
4	Gastric	Oxalate nephropathy	Hypertension
5	Kidney	FSGS (perihilar)	Nephrectomy
6	AL amyloidosis	AL amyloidosis	DM, Hypertension
7	NHL	Infiltration	none
8	Pancreas	MN	none
9	Lung	MCD	none
10	Bladder cancer	FSGS (perihilar)	none
11	MGUS	LCDD	none
12	CLL	LCDD	Hypertension
13	AL amyloidosis	AL amyloidosis	none
14	MGUS	LCDD	none
15	NHL	Infiltration	none
16	MM	LCDD	Hypertension
17	CLL	Infiltration	DM
18	Mesothelioma	FSGS (cellular)	none
19	Renal pelvis	Infiltration	Reduction in size of the contralateral kidney

Non-SACT/non-cancer related AKD accounted for 23.6% (n=13) of the ailments, with the most common diagnosis in this group being diabetic nephropathy (23%, n=3). Glomerulosclerosis was observed in two out of 13 patients (15.4%), as was perihilar focal segmental glomerulosclerosis (15.4%). The remaining 46.2% included isolated cases of IgA nephropathy (7.7%), ciprofloxacin-associated interstitial nephritis (7.7%), ATI (7.7%), post-infectious membranoproliferative glomerulonephritis (7.7%), and oxalate nephropathy linked to vitamin C overuse (7.7%), nephrocalcinosis (7.7%).

Classification in two distinct time periods: comparative results

**Figure 4:**
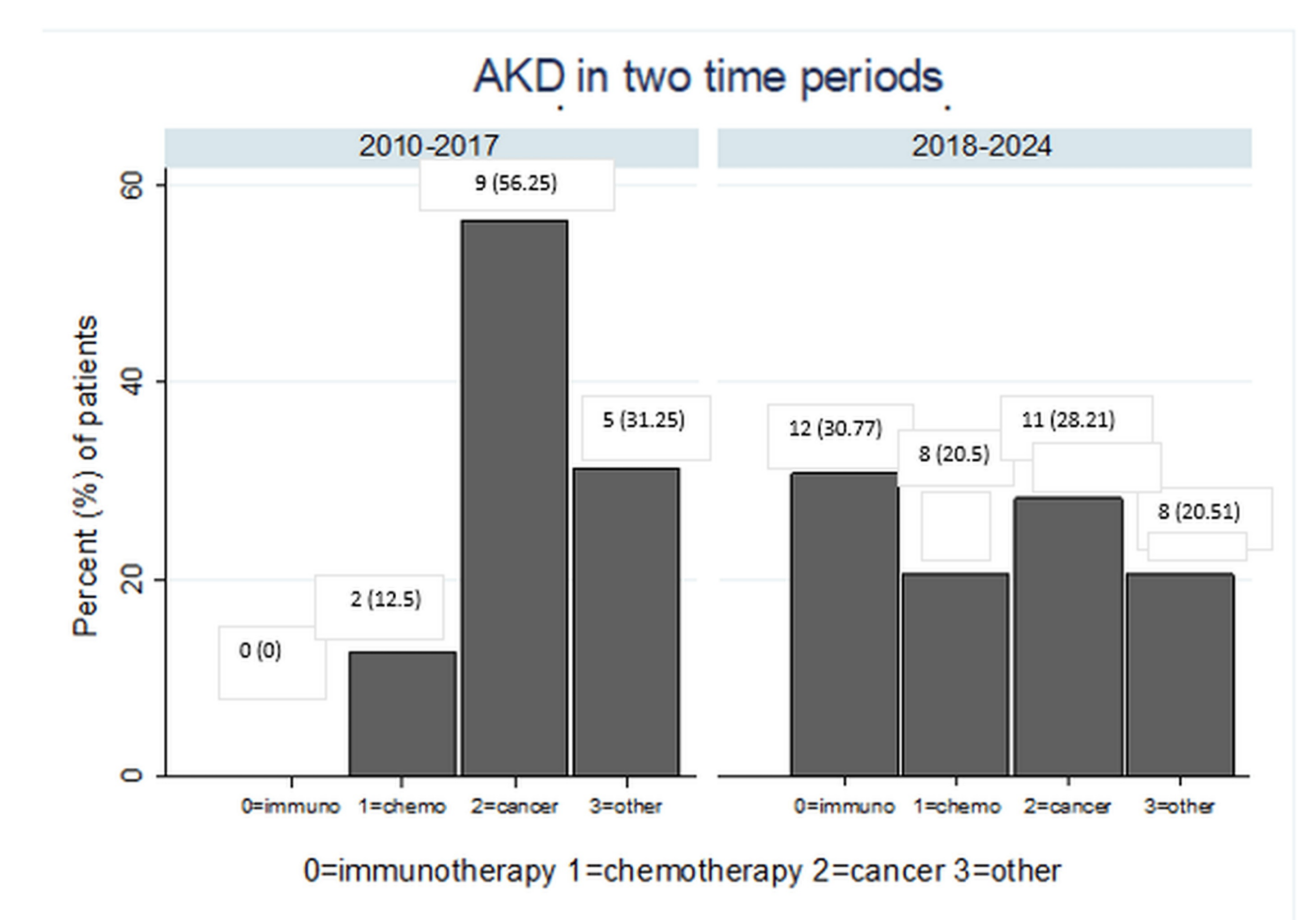
Distribution of acute kidney disease (AKD) etiologies before and after 2017. Values are presented as N (%)

There was a significant increase in systemic anticancer therapy-related kidney injury group when dividing the patients into two subgroups, before and after 2017, which corresponds to the introduction of newer therapies in everyday management of cancer patients. Cancer-related AKD was more frequently observed before 2017, accounting for 56.2% of cases (nine out of 16). In contrast, treatment-related AKD was more commonly detected after 2017, representing 51.2% of cases (20 out of 39) (Fisher’s exact test, p=0.02, Figure 4).

**Figure 5:**
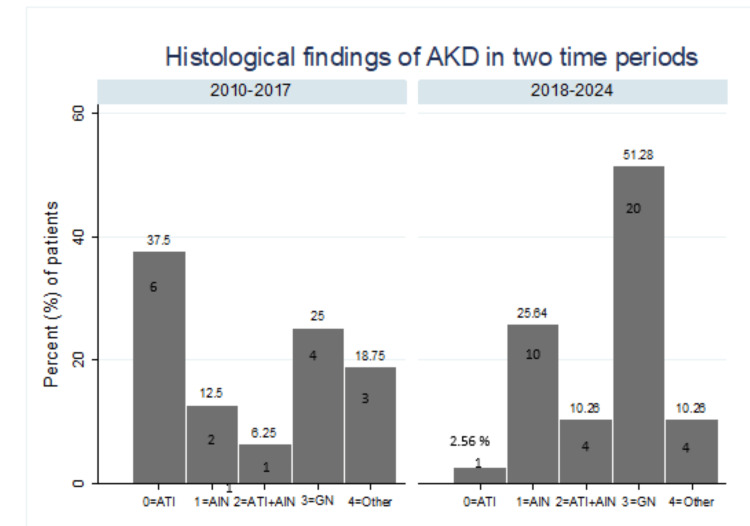
Histopathological findings of AKD before and after 2017. Values are expressed as N (%).

A statistically significant difference in histopathological patterns was also observed between biopsies taken before and after 2017 (Fisher’s exact test, p=0.006). Prior to 2017, ATI was the most common lesion, present in 37.5% of cases (n=6), followed by glomerulonephritis (25%, n=4) and AIN (12.5%, n=2). After 2017, the frequency of glomerulonephritis and AIN increased, occurring in 51.3% (n=20) and 25.6% (n=10) of biopsies, respectively (see Figure 5).

Management and outcomes of AKD

**Figure 6:**
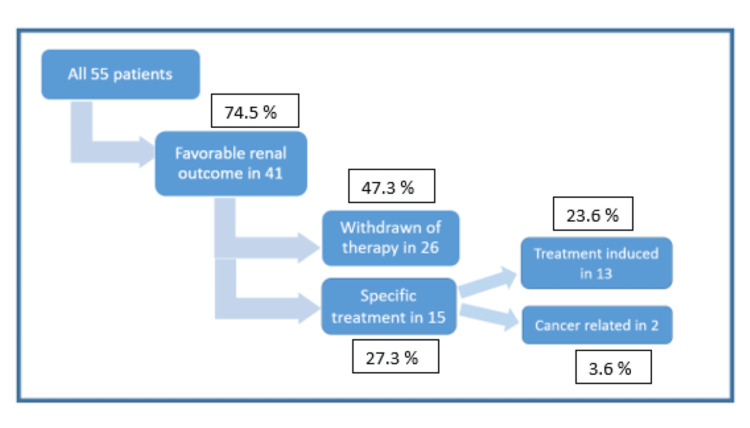
Renal outcomes following kidney biopsy in cancer patients with AKD. Values are presented as number (N) and percentage (%).

A favorable renal outcome was observed in 41 patients (74.5%), occurring after withholding SACT in 47.3% (n=26) or after starting specific therapy with corticosteroids and related anti-inflammatory therapies (CRT) in 27.3% (n=15). CRT included corticosteroids in 80% (n=12), calcineurin inhibitors in 13.3% (n=2) for FSGS and one case of bevacizumab-induced hyaline occlusive glomerulopathy, and colchicine in 6.7% (n=1) for AA amyloidosis (Figure 6).

All patients who received CRT (15/15) recovered from renal injury. In two cases with FSGS and MCD, cancer was considered related to AKD; the remainder were evaluated as SACT-related AKD.

Dialysis was required in six patients (10.9%), with oxalate nephropathy (n=2), ifosfamide toxicity (n=2), LCDD (n=1), and severe infection (n=1) reported. During the observation period, 27 patients (49%) died, including five of the six patients on dialysis (83.3%).

## Discussion

Our study represents a 14-year analysis of kidney biopsies in cancer patients with AKD and assesses the spectrum of histopathological findings in this population. There are only a few published studies presenting data from kidney biopsies in these patients [14,16,20,21].

In recent years, there has been a significant improvement in cancer patients’ survival, primarily due to new anticancer therapies, targeting specific molecules involved in abnormal cancer cell proliferation, such as immunotherapy with ICI, TKIs, and anti-VEGF agents, while traditional chemotherapy remains important. Patients with favorable-prognosis cancers are increasingly considered chronically ill, with prolonged life expectancy, so nephrological management can be similar to the general population [16]. Literature suggests that an increasing number of kidney biopsies are now performed in these patients in the setting of AKD, revealing that the type and management of kidney injury are changing [16,21].

In our cohort, we retrospectively examined all biopsies of patients with solid and hematological malignancies who developed AKD. Focal segmental glomerulosclerosis was the most common glomerulopathy, likely reflecting the age and incidence of comorbidities such as hypertension and diabetes. Interestingly, three out of four patients with sarcoma receiving a combination of ifosfamide and doxorubicin, developed karyomegalic interstitial nephritis, a rare type of interstitial nephritis related to ifosfamide [22]. The drug was withdrawn, but two of them ended up in dialysis, and all three died during the observation period.

Notably, acute interstitial nephritis (AIN), with or without acute tubular injury (ATI), was identified in 30.9% of patients overall; however, among those with SACT-related AKD, the prevalence increased markedly to 82.6%. When patients were stratified into pre- and post-2017 cohorts to reflect the introduction of newer therapies, treatment-related AKD was more common in the post-2017 period, predominantly driven by ICI-associated nephrotoxicity.

As noted in the literature, other lesions beyond AIN can be encountered in treatment-related AKD. In this study, we reported four notable cases of glomerular injury in the context of anticancer therapy-related kidney injury: one case of full-house membranous nephropathy in a patient with non-small cell lung cancer (NSCLC) [13,23] one case of AA amyloidosis in another patient with NSCLC, one case of cellular FSGS in a patient with endometrial cancer, all of whom were receiving pembrolizumab, a PD1 blocking antibody, and one case of hyaline occlusive glomerular microangiopathy in a patient with glioblastoma under bevacizumab (VEGF-A). Notably, two patients who were off treatment, one with excessive vitamin C intake and B-cell Hodgkin lymphoma, and another with surgically treated gastric cancer, exhibited oxalate nephropathy; they both required dialysis.

Beyond documenting histological lesions, the purpose of this study was to assess the clinical importance of kidney biopsy in clinical decision-making, regarding the continuation or adjustment of treatment. Patients who were under SACT at the time of biopsy and kidney injury, evaluated for therapy-related AKD, discontinued therapy. Specific therapy was initiated based on histological findings, leading to improved renal function in all treated patients.

Based on renal histopathology, CRT as a corticosteroid in first-line therapy (12) was initiated in 12 patients with tubulointerstitial injury. One patient with AA amyloidosis received empirical therapy with prednisone combined with colchicine. Two patients with cancer-related glomerulopathies (minimal change disease and FSGS) were treated with cyclosporine, resulting in improved proteinuria. Overall, renal outcomes were favorable in all patients receiving CRT as histology-directed therapy.

The present study has several limitations related to its retrospective design. First, the cohort is relatively small and heterogeneous. Second, cancer stage and metastatic status were not captured. Third, detailed laboratory parameters such as full blood count, eosinophilia or eosinofiluria, and immunological tests were not available, and medication use other than anticancer therapy was not recorded.

In addition, AKD could be assessed only in the biopsied subgroup, as AKD data were not documented for the wider cohort, limiting generalizability. The determination of “SACT-related” AKD relied on routine clinical assessment rather than a standardized causality algorithm, and the documentation was insufficient to apply a structured framework or evaluate inter-reviewer consistency. Definitions of AKD, recovery, and the “favorable outcome” composite were not fully aligned, and incomplete timing data prevented the use of time-to-event approaches. The pre- versus post-2017 comparison could not be adjusted for changes in case-mix or practice, restricting it to descriptive interpretation.

Another limitation is that the apparent benefit of targeted treatment may reflect confounding by indication, as there were no untreated controls and timing data were insufficient for time-aware analyses or outcome curves, constraining interpretation of treatment effects.

Finally, the study did not prespecify a primary endpoint and included multiple unadjusted analyses, limiting the findings to descriptive rather than inferential conclusions. Despite these limitations, this is, to our knowledge, the first study examining AKD in a Greek cancer population, an area with very limited existing data.

## Conclusions

In the field of oncology, survival rates have increased, positioning cancer as a chronic disease rather than a terminal condition. However, AKD remains a frequent and serious complication, necessitating a more proactive nephrological approach, including kidney biopsies. Our study identifies AIN as the most frequent finding, particularly in treatment-related AKD cases, though a broad spectrum of histological lesions beyond AIN and ATI is also observed, and reveals a shift in histological patterns over time. These findings underscore the critical role of biopsy in guiding clinical decisions, with most patients achieving favorable renal outcomes. Further observational, prospective studies are warranted in the field of onconephrology.
